# Editorial: Woody oil crops: key trait formation and regulation

**DOI:** 10.3389/fpls.2023.1328990

**Published:** 2023-11-10

**Authors:** Heping Cao, Wenfang Gong, Jun Rong, Deyi Yuan

**Affiliations:** ^1^ United States Department of Agriculture, Agricultural Research Service, Southern Regional Research Center, New Orleans, LA, United States; ^2^ Key Laboratory of Cultivation and Protection for Non-Wood Forest Trees, Central South University of Forestry and Technology, Changsha, China; ^3^ School of Life Sciences, Nanchang University, Nanchang, China

**Keywords:** woody oil crops, trait formation, molecular mechanisms, regulation, oil content (OC) and fatty acid composition

Woody oil crops are renewable forest resources to produce high-quality oils for food, feed and industrial uses. They contain diverse fatty acids and valuable nutritional components. They can be edible and/or industrial crops (**Table 1**). Edible woody oil crops include cocoa (Medeiros de Azevedo et al., 2020), coconut (Deen et al., 2021), hazelnut (Crews et al., 2005), idesia (Zhang et al., 2023), maple (Song et al., 2022), oil palm (Mancini et al., 2015), oil olive (Battino et al., 2019), pecan (Scapinello et al., 2017), peony (Yang et al., 2017), pine (Zeng et al., 2012), yellow horn (Liang et al., 2021; Zang et al., 2021), oil-tea camellia (Zeng et al., 2014; Luan et al., 2020; Li et al., 2022; Song et al., 2023) and walnut (Rébufa et al., 2022). Industrial woody oil crops include castor (Román-Figueroa et al., 2020), camelina (Berti et al., 2016), crambe (Lalas et al., 2012), flax (Goyal et al., 2014) and Tung (Dyer et al., 2002; Shockey et al., 2006; Cao et al., 2013; Zhang et al., 2014; Li et al., 2017; Liu et al., 2019; Zhang et al., 2019). Many woody oil crops have special fatty acid composition (**Table 1**). Key traits of woody oil crops are essential for breeding and production, such as fruit/seed yield, size, weight, oil content, fatty acid and other valuable compositions, tolerance to drought, cold, and low nutrition stresses. Compared to herbaceous oil crops, key trait formation and regulation in woody oil crops are not well studied.

**Table 1:** Woody oil crops and their oil content and major oil composition.

No.	Common name	Species	Oil content	Major component	References
1	Camelina	*Camelina sativa*	30–47%	30–40% α-linolenic acid, 15–25% linoleic acid	(Berti et al., 2016)
2	Castor	*Ricinus communis*	45–60%	90% ricinoleate	(Román-Figueroa et al., 2020)
3	Cocoa	*Theobroma cacao*	45–60%	58% linoleic acid	(Medeiros de Azevedo et al., 2020)
4	Coconut	*Cocos nucifera*	65–74%	40–50% lauric acid	(Deen et al., 2021)
5	Crambe	*Crambe abyssinica*	25–40%	55–64% erucic acid	(Lalas et al., 2012)
6	Flax	*Linum usitatissimum*	41%	39–60% α-linolenic acid	(Goyal et al., 2014)
7	Hazelnut	*Corylus heterophylla* Fisch.	50–75%	90% oleic and linoleic acids	(Crews et al., 2005)
8	Idesia	*Idesia polycarpa* Maxim.	21–44%	63–71% linoleic acid	(Zhang et al., 2023)
9	Maple	*Acer truncatum* Bunge	42–46%	54% ω-9 and 31% ω-6 fatty acids	(Song et al., 2022)
10	Oil palm	*Elaeis guineensis* Jacq.	50–55%	48% lauric acid	(Mancini et al., 2015)
11	Olive	*Olea europaea*	31–56%	73% oleic acids	(Battino et al., 2019)
12	Pecan	*Carya cathayensis* Sarg.	60–70%	49–77% oleic and 13–40% linoleic acids	(Scapinello et al., 2017)
13	Peony	*Paeonia suffruticosa* Andr	27–33%	>38% α-linolenic acid	(Yang et al., 2017)
14	Pine	*Pinus koraiensis*	58–69%	30% α-terpineol, 24% linalool, 17% limonene,15% anethole	(Zeng et al., 2012)
15	Tea-oil tree	*Camellia oleifera Abel*	47–60%	80% oleic acids	(Zeng et al., 2014; Luan et al., 2020; Li et al., 2022; Song et al., 2023)
16	Tung	*Vernicia fordii and Vernicia montana*	50–60%	77–80% α-eleostearic acid	(Dyer et al., 2002; Shockey et al., 2006; Cao et al., 2013; Zhang et al., 2014; Li et al., 2017; Liu et al., 2019; Zhang et al., 2019)
17	Walnut	*Juglans regia*	50–70%	50–74% linoleic acid	(Rébufa et al., 2022)
18	Yellow Horn	*Xanthoceras sorbifolium Bunge*	50–60%	28–41% linoleic acid and 27–42% oleic acid	(Liang et al., 2021; Zang et al., 2021)

This Research Topic is aimed to summarize recent advances in key trait formation and regulation in woody oil crops for facilitating breeding and production. Thirteen articles have been published including 12 original research articles and one review article. Among them, ten papers focus on *Camellia oleifera*, and one each on *Pinus koraiensis*, *Paeonia ostii* and *Carya cathayensis*.

## Genome and genetic diversity

*Camellia oleifera* genome is very complex. One article reviewed the “*Genomic and genetic advances of oiltea-camellia (Camellia oleifera)*” (Ye et al.). The report summarized the recent assembly of the reference genomes and identified putative genes related to economic traits, disease resistance and environmental stress tolerances. To explore the genetic diversity of wild *C. oleifera* phenotypic traits, another article reported “Characterization and comprehensive evaluation of phenotypic characters in wild *Camellia oleifera* germplasm for conservation and breeding” (Chen et al.). They used 143 wild *C. oleifera* germplasm resources and identified 41 characters based on the quantization of physical and chemical descriptors and digital image analysis.

## Flower bud formation

The number of flower buds is a main factor affecting the crop yield. One investigation studied “*Co-regulatory effects of hormone and mRNA–miRNA module on flower bud formation of Camellia oleifera”* (Du et al.). The results showed that GA_3_, ABA, tZ, JA, and SA contents in the buds were higher than those in the fruit and that differentially expressed genes were notably enriched in hormone signal transduction and the circadian system.

## Oil accumulation

*Camellia oleifera* oil quality is mainly determined by linoleic acid (LA) and α-linolenic acid (ALA) content. One study reported “*Enhancing the accumulation of linoleic acid and α-linolenic acid through the pre-harvest ethylene treatment in Camellia oleifera”* (Li et al.). The study confirms the role of ethylene in LA and ALA regulation and provides new insights into the potential utilization of ethylene as a LA and ALA inducer.

## Nutrient deficiency

Phosphorus deficiency in the acidic soil poses severe challenges for the growth and productivity. WRKY transcription factors play important roles in plant responses to biotic/abiotic stresses. One article reported “*Genome-wide identification of the WRKY gene family in Camellia oleifera and expression analysis under phosphorus deficiency*” (Su et al.). The authors identified 89 WRKY proteins into three groups, detected WRKY variants and mutations, and suggested that WRKYs play a crucial role in the transportation and recycling phosphate in leaves.

## Cold and drought stresses

The molecular mechanisms of freezing tolerance are unresolved in perennial trees. One investigation found that “*Field plus lab experiments help identify freezing tolerance and associated genes in subtropical evergreen broadleaf trees: A case study of Camellia oleifera”* (Xie et al.). Combing transcriptome results from the field and lab experiments, the common genes associated with freezing-stress responses were identified. Drought stress is another major obstacle in *C. oleifera* planting industry. The other investigation reported that “Integration of mRNA and miRNA analysis reveals the differentially regulatory network in two different *Camellia oleifera* cultivars under drought stress” (He et al.). Their research improves the understanding of the regulatory network response to drought stress and variety-specific responses improving drought tolerance.

## Grafting

*Camellia oleifera* scion significantly affects the rootstock properties after grafting and impacts the grafted seedling growth. One study reported “*Untargeted metabolism approach reveals difference of varieties of bud and relation among characteristics of grafting seedlings in Camellia oleifera”* (Long et al.). They detected 554 metabolites significantly different among four varieties and 29 metabolic pathways significantly changed by metabolomics analysis.

## Disease resistance

Anthracnose outbreak severely affects oil tea camellia production in China. One paper revealed that “*Overexpression of dihydroflavonol 4-reductase (CoDFR) boosts flavonoid production involved in the anthracnose resistance*” (Yang et al.). The results showed that *CoDFR* may play an important role in flavonoid-mediated defense mechanisms during anthracnose invasion in resistant *C. oleifera*. WRKY transcription factor family members are vital regulators in plant response to pathogen infection. Another paper reported “*Identification of Camellia oleifera WRKY transcription factor genes and functional characterization of CoWRKY78*” (Li et al.). They identified 90 WRKY members, verified the expression patterns between anthracnose-resistant and -susceptible cultivars, and demonstrated that multiple candidate *CoWRKY*s can be induced by anthracnose.

## Oil synthesis gene

Chinese hickory (*Carya cathayensis*) produces nuts with high-quality edible oils rich in oleic acid. Stearoyl-ACP desaturase (SAD) plays an important role in oleic acid accumulation by catalyzing the first step converting stearic acid to oleic acid. One paper reported the “*Analysis of Delta(9) fatty acid desaturase gene family and their role in oleic acid accumulation in Carya cathayensis kernel*” (Si et al.). The study identified five members of *SAD* genes, isolated the full-length cDNAs, analyzed their expression, located them in the chloroplast, and studied their function in *Saccharomyces cerevisiae*, *Nicotiana benthamiana*, and walnut.

## N and P nutrients

Pine (*Pinus koraiensis*) produces high-quality timber and high value health-care nut oil. One article reported that “*Large investment of stored nitrogen and phosphorus in female cones is consistent with infrequent reproduction events of Pinus koraiensis, a high value woody oil crop in Northeast Asia*” (Wu et al.). High nutrient sink strength of cones and vegetative tissues of reproductive branches suggested that customized fertilization practices can help improve crop yield in *Pinus koraiensis*.

## Non-structural carbohydrates

Non-structural carbohydrates (NSC) play important roles in energy supply for normal growth and reproduction under environmental stress. One article described the “*Effects of NSC in different organs and at different growth stages on the yield of oil peony Fengdan with different ages*” (Wang et al.). Results showed that the biomass, yield (seed biomass), soluble sugars, starch, and NSC reserve at the whole tree level increased with the increase in age. NSC level, particularly the concentration of soluble sugars in stems mainly influences Fengdan yield.

## Author contributions

HC: Writing – original draft, Writing – review & editing, Conceptualization, Project administration. WG: Writing – original draft. JR: Writing – review & editing. DY: Writing – review & editing.
